# A Systematic Review of the Effects of Aromatherapy with Lavender Essential Oil on Depression

**DOI:** 10.5195/cajgh.2020.442

**Published:** 2020-03-31

**Authors:** Azar Jafari-Koulaee, Forouzan Elyasi, Zohreh Taraghi, Ehteram Sadat Ilali, Mahmood Moosazadeh

**Affiliations:** 1 Student Research Committee, Mazandaran University of Medical Sciences, Sari, Iran; 2 Department of Psychiatry, School of Medicine, Mazandaran University of Medical Sciences, Sari, Iran; 3 Addiction Institute, Mazandaran University of Medical Sciences, Sari, Iran; 4 Department of Geratric Nursing, School of Nursing and Midwifery, Mazandaran University of Medical Sciences, Sari, Iran; 5 Health Science Research Center, Mazandaran University of Medical Sciences, Sari, Iran

**Keywords:** Aromatherapy, Lavender, Depression, Complementary medicine

## Abstract

**Introduction:**

Depression is considered as one of the most serious health issues worldwide, and the search for the most effective and safe treatments for depression is essential. Aromatherapy with lavender have attracted the attention of many researchers due to their low cost and ease of use, so this study was conducted to review of the effects of aromatherapy with lavender essential oil on depression.

**Methods:**

This systematic review study was conducted by searching the databases (SID, Magiran, Google-Scholar, Medline via PubMed, Scopus, and Web of Science) by using the keywords such as depression, Lavandula, Lavender, and Aromatherapy, as well as applying OR and AND operators to the end of January 1, 2020 A.D. The inclusion criteria were: 1) Interventional studies that determined keywords were in the title or keywords of the article, 2) aromatherapy was conducted through inhalation or massage, 3) the full text of paper was accessible, and 4) published in English or Persian. Finally, the information obtained from articles was extracted using a checklist.

**Results:**

Out of 278 studies, 9 studies were included to the systematic review process after screening and eliminating duplicate papers according to the purpose of the study. Aromatherapy with lavender essential oil was conducted on the patients suffering from migraine, patients with the acute coronary syndrome, patients undergoing hemodialysis, community-dwelling older adult, and postpartum depression. The results obtained from some studies showed the positive effect of aromatherapy with lavender essential oil on depression whereas some studies did not report the effect of aromatherapy with lavender on depression significantly.

**Conclusions:**

It seems that aromatherapy with lavender probably can be used as a complementary, simple, and inexpensive method to improve mild and moderate depression. It is recommended to earmark using a collaborative approach and make use of interdisciplinary and psychology specialists as well as complementary medicine in applying aromatherapy with lavender essential oil.

Depression is considered as one of the most serious healthcare problems, and the statistics of individuals suffering from it is increasing. According to statistics, over 264 million suffer from depression throughout the world. ^1^ In a review study, the prevalence of depression in Iran was reported 6% to 73%.^2^ The pathogenesis of depression is complicated, and numerous risk factors can impact depression affection. These factors include the medical chronic condition, stress, chronic pain, familial history, female gender, economic conditions, joblessness, drug abuse, low self-esteem, lack of social support, marital status, brain injury, and age.^3^-^6^ Furthermore, obesity, malnutrition, physical inactivity, lack of sunlight, lack of sleep, and social problems in modern societies influence the rate of depression affection.^6^ Unfortunately, suffering from depression is followed by unpleasant consequences; for example, a decrease in a patient's performance in personal, social, and familial dimensions and even suicide.^7^,^8^ Indeed, depression is the source of many somatic disorders,^9^,^10^ insomnia,^11^ sexual disorders,^12^,^13^ and disorders in biologic rhythms.^14^ Hence, it seems necessary to seek the best, most effective, and least hazardous therapies and approaches to improve depression.

In this respect, numerous pharmacological and non-pharmacological therapies are offered for depression improvement.^15^,^16^ Concerning the pharmacological therapies, it seems that many drugs play significant roles in treating psychological disorders; however, with respect to the presence of many complaints regarding the uselessness of these drugs for all patients, along with the emergence of diverse side effects and tolerance (if they are used in the long-run), numerous researchers have noticed using non-pharmacological therapies.^17^-^19^ Several studies have addressed the positive effect of some non-pharmacological therapies, including art therapy,^20^ music,^21^,^22^ and aromatherapy,^23,24^ on the improvement in depression and anxiety. In the meantime, aromatherapy, as a non-pharmacological method, has been exploited in many studies owing to its hazardless and convenient usage.^25^ Aromatherapy employs the fragrant oils extracted from flowers and herbs to treat varying diseases. Essential oils can be used by inhaling, taking bath, or during massages. ^25^,^26^ Lavender is of those herbs that are used in aromatherapy.^27^ This herb is from the lamiaceae family with the scientific name of lavandula angustifolia.^28^ Many studies have addressed the anti-pain,^29^,^30^ antianxiety and anti-depressant,^27^,^31^,^32^ and sleep improvement^33^ effects. Also, some researchers have believed that lavender exerts its psychological effects through the effects on the limbic system, especially the amygdala and hippocampus. Mechanism of this plant on the cell surface is not completely known, but it has been reported that this plant probably had a similar function to benzodiazepines and increased GABA (gamma aminobutyric acid) in the amygdala.^34^ On the other hand, the review of the available databases showed that although some studies have reported the positive effects of aromatherapy with lavender on depression,^27^,^35^-^40^ but some others have reported reverse results.^41^,^42^ Thus, considering the presence of contradictions in this field, lavender's application to improving depression is still being argued. Hence, to access more precise and comprehensive evidence, this study reviewed the effect of aromatherapy with lavender on depression.

## Methods

This systematic review study was conducted based on the Preferred Reporting Items for Systematic Reviews and Meta-Analysis (PRISMA)^43^ in 2020. To review the studies, we considered the components of the Population Intervention Comparison Outcome (PICO) structured review as a part of the research process.^44^ PICO is one of the most suitable methods in discovering different literature for systematic review studies regarding diseases, therapeutic interventions, and consequences.^45^ At first, some factors, including the intervention type, kind of applied study, titles, and databases of concern are inserted in the standard PICO checklist for initial information estimation. To search more comprehensively, we searched the informational databases of SID, Magiran, Google-Scholar, Medline via PubMed, Scopus, and Web of Science by using the Mesh and non-Mesh keywords such as depression, Lavandula, Lavender, and Aromatherapy, as well as applying OR and AND operators, with no time limitation since establishment of databases to the end of January 1, 2020 A.D. Also, the final references of searched articles, were evaluated as additional resources. The details of how keywords and operators were used to search the database are described below:

- Scopus:(TITLE-ABS-KEY (lavender) OR TITLE-ABS
  KEY (lavandula) AND TITLE-ABS-
  KEY (aromatherapy) AND TITLE-ABS-KEY (depression))
- WOS:(TS=(aromatherapy AND (lavender OR lavandula) AND depression)) AND LANGUAGE: (English OR Persian) AND DOCUMENT TYPES: (Article)
- PubMed:(((Lavender[Title/Abstract]) OR Lavandula[Title/Abstract]) AND Aromatherapy[Title/Abstract]) AND Depression[Title/Abstract]
- Google Scholar:(Lavender OR Lavandula) AND Aromatherapy AND Depression
- SID:(Lavender OR Lavandula) AND Aromatherapy AND Depression

**Figure 1.**
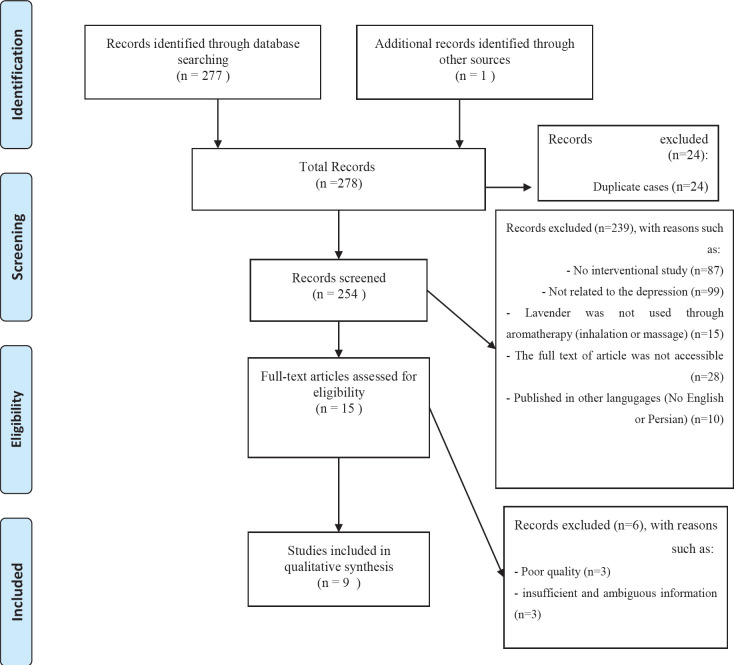
Flowchart of article selection using PRISMA

Two researchers (A.J. & E.I.) reevaluated and reinvestigated the resources and databases to warrant the sufficiency and inclusiveness of information and article searching. After primary screening and removing duplicate cases, the articles were reviewed on the basis of inclusion and exclusion criteria, and finally eligible articles were included in the systematic review process.

The inclusion criteria were: 1) Interventional studies that determined keywords were in the title or keywords of the them, 2) aromatherapy was conducted through inhalation or massage, 3) the full text of article was accessible, and 4) published in English or Persian. The studies with poor qualities, insufficient and ambiguous information were excluded from the process.

The quality evaluation of the studies was conducted by the JADAD checklist. The JADAD checklist includes the three items of randomization, blinding, and withdrawals and dropouts that are in direct relationship with bias control in interventionist studies. The quality of studies was ranked as low-quality and high-quality studies if they were scored 1-2 and 3-5, respectively. ^46^,^47^ In the present study, we eliminated the scores that were below 3 based on the checklist after the quality evaluation, and the scores above 3 were included in the review process.

Finally, a checklist was used to extract the information of the papers imported to the review. This checklist consisted of items such as the author's name, year of publication, type of study, sample size, type of variable, instrument, type of intervention, and results.

## Results

Out of 278 studies, 9 studies were included to the systematic review process after screening and eliminating duplicate articles according to the purpose of the study. The details related to the stages of study selection are represented in Figure 1.

The intervention in these studies was inhalation aromatherapy ^27^,^36^-^42^ or aromatherapy massage ^35^,^40^ with lavender. Aromatherapy with lavender essential oil was conducted on the patients suffering from migraine,^41^ patients with the acute coronary syndrome,^35^ patients undergoing hemodialysis,^27^,^39^ community-dwelling older adults,^36^,^40^ and postpartum depression.^37^,^38^,^40^ Depression was measured by different scales, including Hospital Anxiety and Depression Scale (HADS), Geriatric Depression Scale (GDS), Beck Depression Inventory (BDI), Depression Anxiety Stress Scales-21, Center for Epidemiological Studies Depression (CES-D) Scale, and Edinburgh Anxiety Questionnaire and Stress Scale. The results obtained from studies showed the positive effect of aromatherapy with lavender essential oil on depression,^27^,^35^^-^^40^ whereas some studies did not report the positive effect of aromatherapy with lavender on depression significantly.^41^,^42^ Other details are presented in Table 1.

**Table 1.** Summary of data extracted from the reviewed articles (N=9)

Author (year)	Study design	Sample size (n) / Type of sample of the study	Type of intervention / Follow-up period	Variable/ Instruments	Results	Conclusion	Quality of study
Bagheri-Nesami1 et al. (2017)	Randomized control trial	ni=36; nc=36 / Hemodialysis patients	Intervention group: aromatherapy with 3 drops of lavender essential oil 5% for 10 minutes every time they underwent hemodialysis for a period of one month Control group: routine care Follow-up period: 4 weeks	Anxiety and depression/ Hospital Anxiety and Depression Scale (HADS)	Before intervention (control group): Mean±SD= 4.05 ±4.48 Two weeks after intervention (control group): Mean±SD= 5.00 ± 5.13 Four weeks after intervention (control group): Mean±SD= 4.27 ± 5.04 p = 0.221 Before intervention (experimental group): Mean±SD= 4.54 ± 4.11 Two weeks after intervention (experimental group): Mean±SD= 3.11 ± 3.06 Four weeks after intervention (experimental group): Mean±SD= 3.82 ± 4.07 p = 0.019 Between the two groups: p = 0.005	Significant differences existed between the two groups with respect to depression levels before, two and four weeks after the intervention.	3
Tayebi et al. (2015)	Randomized control trial	ni=30; nc=30 / Hemodialysis patients	Interventional group: Inhale the lavender essential oil smeared on a piece of cloth (three drops of oil) for one hour during the hemodialysis Control group: routine care Follow-up period: 4 weeks	Depression, anxiety, stress / Depression Anxiety Stress Scales-21	Pre-test (interventional group): Mean±SD= 6.7±4.9 Pre-test (control group): Mean±SD= 7.4 ± 6.9 Post-test (interventional group): Mean±SD= 4.9±5.55 Post-test (control group): Mean±SD= 7.4 ± 7.3 Between the two groups: p<0.001	Aromatherapy with lavender essential oil might reduce depression and stress among hemodialysis patients.	3
Bahrami et al. (2016)	Randomized control trial	ni=45; nc=45 / Patients with acute coronary syndrome	Intervention group received reflexology with lavender essential oil Control group: routine care Follow-up period: 4 weeks	Depression / Hospital's Anxiety and Depression Scale	Pre-test (experimental group): Mean±SD = 12.51 ± 5.40 Pre-test (control group): Mean±SD = 8.04 ± 4.71 p = 0.439 Post-test (experimental group): Mean±SD = 8.04 ± 4.71 Post-test (control group): Mean±SD = 11.11 ± 3.42 p = 0.001	Aromatherapy massage can be considered by clinical nurses an efficient therapy for alleviating psychological and physiological responses among older women suffering from acute coronary syndrome.	3
Xiong et al. (2017)	Randomized control trial	ni=20; ni=20; nc=20 / Chinese Community-Dwelling Older Adults	Aromatherapy massage group: 30 min of aromatherapy massage with 5ml oil lavender (diluted in sweet almond oil to a concentration of 1%), twice weekly for 8 weeks Aromatherapy inhalation group: 30 min of nasal inhalation of 50ml of the compound essential oils blended in 10ml of purified water, twice weekly for 8 weeks Control group: no intervention Follow-up period: 10 weeks	Depression/ Geriatric Depression Scale Short Form (GDS-SF) & Patient Health Questionnaire-9 (PHQ-9)	Pre-test (massage group): Mean±SD = 6.70 ± 1.92 Pre-test (inhalation group): Mean±SD = 7.00 ± 1.34 Pre-test (control group): Mean±SD = 6.80 ± 1.47 Post-test (massage group): Mean±SD = 3.25 ± 2.15 Post-test (inhalation group): Mean±SD = 3.75 ± 1.68 Post-test (control group): Mean±SD = 6.65 ± 1.23 6-week follow-up (massage group): Mean±SD = 3.95 ±2.07 6-week follow-up (inhalation group): Mean±SD = 4.35 ±1.56 6-week follow-up (control group): Mean±SD = 6.95 ±1.25 10-week follow-up (massage group): Mean±SD = 3.63 ±2.00 10-week follow-up (inhalation group): Mean±SD = 4.55 ±1.29 10-week follow-up (control group): Mean±SD = 6.70 ±1.61	After intervention, the aromatherapy massage and inhalation groups demonstrated significantly lower GDS-SF than control participants.	3
Janizadeh et al. (2016)	Quasi experimental	ni=20; ni=20; ni=20; nc=20 / Elderly Women	Interventional group 1: yoga practice Interventional group 2: yoga practice combined with lavender use in the first month of training Interventional group 3: yoga practice combined with lavender use in the second month of training All three interventional groups practiced yoga for 2 months. Control group: routine care Follow-up period: 8 weeks	Depression/ Beck Depression Inventory (BDI)	Pre-test (interventional group 1): Mean±SD= 24.40 ±12.72 Post-test (interventional group 1): Mean±SD= 21.20 ± 12.12 p= 0.001 Pre-test (interventional group 2): Mean±SD=23.10±11.77 Post-test (interventional group 2): Mean±SD= 14 ±7.65 p= 0.001 Pre-test (interventional group 3): Mean±SD=19.50±8.42 Post-test (interventional group 3): Mean±SD= 13.40 ± 7.73 p= 0.001 Pre-test (control group): Mean±SD= 21.30±8.55 Post-test (control group): Mean±SD= 20.80± 8.63	Greater reduction in depression in the combined groups compared to the yoga practice group. However, there was no significant difference between the two combined groups	3
Jafari-Koulaee et al. (2018)	Randomized control trial	ni=30; nc=30 / Migraine patients	Interventional group: Inhaled 2-3 drops of lavender essence for 15 min, three times a week for 4 consecutive weeks Control group: routine care Follow-up period: 4 weeks	Depression / Beck Depression Inventory (BDI-13)	Pre-test (interventional group): Mean±SD= 10.93±0.56 Pre-test (control group): Mean±SD= 9.70±2.33 Post-test (interventional group): Mean±SD= 5.23±0.57 Post-test (control group): Mean±SD= 5.10 ± 0.53 p=0.13	Lavender essential oil can be useful for reducing depression and headache disability in migraine patients.	3
Kianpour et al. (2016)	Randomized control trial	ni=25; nc=34; np=31 / Women in the postpartum period	Interventional group: 7 drops of lavender oil and 1cc rose water at the concentration of 100%,	Postpartum Depression / Edinburgh questionnaire	Before intervention (interventional group): Mean±SD= 10.37 ±3.21 Before intervention (control group): Mean±SD= 10.61±4.21 Before intervention (placebo group): Mean±SD= 9.71 ±4.20	The use of aromatherapy can be recommended in high-risk women.	4
			Placebo group: 7 drops of odorless sesame seed oil, with 1 cc of musk willow sweat at the concentration of 100% Control group: routine care Follow-up period: 6 weeks		p>0.05 2 weeks after delivery (interventional group): Mean±SD= 7.80 ±3.90 2 weeks after delivery (control group): Mean±SD= 8.51 ±3.61 2 weeks after delivery (placebo group): Mean±SD= 11.70±4.10 p>0.001 6 weeks after delivery (interventional group): Mean±SD= 6.80 ±3.61 6 weeks after delivery (control group): Mean±SD= 9.50 ±3.03 6 weeks after delivery (placebo group): Mean±SD= 7.90 ±3.30 p=0.01		
Kianpour et al. (2016)	Randomized control trial	ni=70; nc=70 / Women in the postpartum period	Interventional group: Aromatherapy consisted of inhaling three drops of lavender essential oil every 8h with for 4 weeks Control group: no aromatherapy Follow-up period: 12 weeks	Postpartum Depression / 21-item Depression, Anxiety, and Stress Scale and the Edinburgh stress, anxiety, and depression scale	2 weeks after intervention (interventional group): Mean±SD=5.31±4.42 2 weeks after intervention (control group): Mean±SD= 7.34 ±5.16 p=0.003 1 month after intervention (interventional group): Mean±SD= 4.10 ±3.92 1 month after intervention (control group): Mean±SD= 7.59 ±5.14 p < 0.0001 3 months after intervention (interventional group): Mean±SD= 3.81 ±3.48 3 months after intervention (control group): Mean±SD= 7.27 ±5.11 p < 0.0001	Inhaling the scent of lavender for 4 weeks can prevent stress, anxiety, and depression after childbirth.	3
Sehhatie et al. (2015)	Controlled double-blinded random clinical trial	ni=158; nc=162/ Women in the postpartum period	Interventional group: showering, being in upright posture, aromatherapy with lavender (1 ml solution of	Mothers’ Postpartum Depression / Edinburgh questionnaire	Pre-test (experimental group): Mean±SD= 6.1±3.2 Post-test (experimental group): Mean±SD= 7.8±4.6 Pre-test (Control group): Mean±SD= 6.3±3.2 Post-test (Control group):	The results show the decrease of depression in the intervening group as compared to	4
			20% lavender essence), and soft music without words Control group: the interventions were customary according to delivery interventions. Follow-up period: 8 weeks		Mean±SD= 8.8±5.4 Mean difference (95% CI) = (-0.2-1.8)-0.8 p=0.124	the control group which could be due to the effect of nonpharmacologi cal methods of pain relief in labor.	

## Discussion

The present study aimed to review the effect of aromatherapy with lavender on depression. In this regard, the results of the review showed that the depression of patients undergoing hemodialysis significantly improved in the study of Tayebi et al.^39^ compared to the study of Bagheri Nesami et al.^27^ Perhaps, one of the possible reasons for the difference between the results of these two studies is the duration of every aromatherapy session; i.e., a session lasted one hour in the study of Tayebi et al.,^39^ while it was 15 minutes in the study of Bagheri Nesami et al.^27^ Longer aromatherapy periods may have better effects on depression reduction. Of course, the presence of other factors such as differences in the personal and cultural characteristics of individuals, the patients’ inclinations to use complementary medicine, and many other cases should be considered.

Xiong et al.,^40^ in their study on older adults, reported that depression significantly decreased in the inhalation and massage aromatherapy group compared to the control group. Furthermore, it was observed that the long-term effects of aromatherapy with lavender on depression reduction lasted for 10 weeks after the intervention.^38^ In line with the mentioned study, Kianpour et al.^37^,^38^ conducted a study on postpartum depression that the long-tern effect of aromatherapy with lavender on depression reduction was found 6 weeks, one month, and even three months after delivery. On the other hand, in another study,^49^ although the positive effects of aromatherapy lasted up to six weeks after the intervention, there was not a significant difference between the experimental and control groups in their depression scores after 10 weeks. Perhaps, one of the possible reasons for the difference in results is that the patients of this study^49^ were suffering from cancer, and the kind of aroma applied to aromatherapy was not specified, whereas the participants in the study of Xiong et al.^40^ and Kianpour et al.^37^,^38^ were the community dwelling older adult and women in postpartum period, respectively, and they utilized lavender essential oil.

In a study conducted on postpartum depression, Kianpour et al.^37^,^38^ found that women's depression significantly decreased after aromatherapy with lavender. However, Sehatti et al.^42^ reported that aromatherapy with lavender essential oil did not significantly decrease the depression of women after their delivery. One of the reasons for the difference between results may be the kind of utilized lavender essential oil. Kianpour et al.^37^ and another study^38^ employed a pure lavender essential oil and the one diluted by rose essential oil, respectively, while Sehatti et al.^42^ employed lavender essential oil 20% diluted by distilled water. Moreover, the difference in the time of depression measurement may be another reason. It is because Kianpour et al.^37^ and another study^38^ measured depression 2, 4, and 12 weeks and 2 and 6 weeks after delivery, respectively. However, in another study, depression was assessed 8 weeks after delivery.

Janizadeh et al.^36^ showed that if the lavender essential oil were applied along with yoga, it would have positive effects on the depression reduction of depressed women. Therefore, we can state that aromatherapy with Lavender essential oil combined with other nonpharmacological interventions that impact depression, including yoga, may be more effective in improving depression. Of course, it is worth to mention that some factors such as the type and severity of depression, as well as individuals’ personal and physical conditions and inclinations, should be taken into account. Hence, the conduction of more large-scale studies is necessary for acquiring more comprehensive and precise evidence in this regard.

Overall, the possible reasons for the differences in the results of the studies in terms of severity of decreasing depression may be related to the differences in definition of cases, randomization, blinding and sample size determination. In the reviewed studies, participations were not homogeneous (migraine patients,^41^ acute coronary syndrome patients,^35^ hemodialysis patients,^27^,^39^ community-dwelling older adults,^36^,^40^ and postpartum depression).^37^,^38^,^40^ Also, randomization was done in almost all studies,^27^,^35^^-^^42^ whereas blinding was performed in only two studies.^38^,^42^ On the other hand, although according to the review of the studies, sample size calculation in most of the studies was done based on the sample size formula or an acceptable method for estimating the sample size, but sample size in some of the studies was small or moderate thus, it seems that further large clinical trials with larger sample size are needed to provide more accurate evidence.

The results of the most studies revealed that probably, lavender aromatherapy can improve the depression of patients undergoing hemodialysis, patients suffering from acute coronary syndrome, community dwelling older adults, and postpartum depression. Furthermore, the results showed that probably, aromatherapy can improve depression if it is mixed with other non-pharmacological interventions, including physical exercises and yoga, but available studies are not sufficient. Although, it seems that lavender aromatherapy as a complementary, simple, and inexpensive method can be used by clinical nurses to improve depression (mild to moderate), along with other measures, in patients with chronic diseases or undergoing diagnostic and therapeutic measures and postpartum depression, but further high-quality RCTs studies are needed to confirm these findings and to achieve the best level of evidence in this field. High-quality RCTs studies can provide more reliable findings so that we can use their findings in evidence-based practice. Also, a collaborative and interdisciplinary approach is recommended for applying lavender aromatherapy.

Despite the strengths of this study, one of the limitations of this study is inaccessibility to some of the studies because of publishing in non-English or non-Persian language. A relatively low sample size in some of the studies and lack of high-quality RCTs are other limitations. Meanwhile, the impossibility of meta-analysis due to the heterogeneity of the design of studies and research population is another limitation of the study.

Depression is a major contributor to the overall global burden of disease, and it is essential to support those who are suffering from this mental disorder by applying easy and safe methods. In this regard, it is recommended that future research be conducted focusing on the evaluation of the effect of complementary medicine interventions (e.g. aromatherapy) and the importance of applying them in combination with other interventions to reduce depression and its psychological and social burden of this debilitating disease in the world.
