# Erratum to: *A Systematic Review of the Effects of Aromatherapy with Lavender Essential Oil on Depression*

**DOI:** 10.5195/cajgh.2020.546

**Published:** 2020-03-31

The editors of the Central Asian Journal of Global Health regretfully published the article “A Systematic Review of the Effects of Aromatherapy with Lavender Essential Oil on Depression” with a misspelled word in the title. This mistake has been corrected. The original article has been updated to reflect this change.
